# The Association between Metabolic Syndrome or Chronic Kidney Disease and Hearing Thresholds in Koreans: The Korean National Health and Nutrition Examination Survey 2009-2012

**DOI:** 10.1371/journal.pone.0120372

**Published:** 2015-03-20

**Authors:** Seok Hui Kang, Da Jung Jung, Kyu Hyang Cho, Jong Won Park, Kyung Woo Yoon, Jun Young Do

**Affiliations:** 1 Division of Nephrology, Department of Internal Medicine, Yeungnam University Hospital, Daegu, Republic of Korea; 2 Department of Otorhinolaryngology-Head and Neck Surgery, School of Medicine, Kyungpook National University Hospital, Daegu, Republic of Korea; School of Public Health, Zhejiang University, CHINA

## Abstract

**Background:**

The aim of this study was to determine whether metabolic syndrome (MetS) or chronic kidney disease (CKD) is associated with hearing thresholds in the general Korean population.

**Patients and Methods:**

A total of 16,554 participants were included in this study. MetS was defined using the National Cholesterol Education Program Adult Treatment Panel III guidelines, and CKD was defined as an estimated glomerular filtration rate <60 mL/min/1.73 m^2^ or a dipstick proteinuria result of ≥1+. The hearing thresholds were measured at 0.5, 1, 2, 3, 4, and 6 kHz. Low-frequency (Freq) was defined as pure-tone averages at 0.5 and 1 kHz, while Mid-Freq and High-Freq were defined as the average thresholds at mid-frequency (2 and 3 kHz) and high frequency (4 and 6 kHz), respectively.

**Results:**

In men, the hearing thresholds were 15.1 ± 14.5 dB, 22.2 ± 21.3 dB, and 37.3 ± 26.5 dB for Low-, Mid-, and High-Freq, respectively. In women, the hearing thresholds were 14.9 ± 15.3 dB, 16.6 ± 18.0 dB, and 26.1 ± 21.5 dB for Low-, Mid-, and High-Freq, respectively. The hearing thresholds for men were significantly higher than the hearing thresholds for women in all 3 threshold categories. Male and female subjects with MetS or CKD had higher hearing thresholds than the subjects that did not have these disorders. In the multivariate analysis, MetS was associated with increased hearing thresholds in women, and CKD was associated with increased hearing thresholds in men and women.

**Conclusion:**

MetS is associated with hearing thresholds in women, and CKD is associated with hearing thresholds in men and women. Therefore, patients with MetS or CKD should be closely monitored for hearing impairment.

## Background

Hearing loss significantly affects quality of life [1]. In a recent study, Agrawal et al. reported that the prevalence of hearing loss was 43% and 20% in men and women, respectively, among subjects who were 60–69 years old [2]. In addition, it is anticipated that hearing loss will become a major public health concern as the number of elderly individuals increase. Although the pathogenesis of hearing loss is not well understood, recent studies suggest that vasculopathies in the stria vascularis play a key role in cochlear dysfunction [3]. In addition, atherosclerosis due to metabolic factors can induce impaired homeostasis in the cochlear blood supply [4], and the resulting hypoxia can increase oxidative stress and damage to the cochlear cells. This hypothesis suggests that metabolic factors contribute to sensorineural hearing loss and presbycusis.

The incidence of metabolic syndrome (MetS) and chronic kidney disease (CKD) has increased significantly over the last decade, and it is expected to continue rising [5–8]. MetS consists of five metabolic disturbances, including impaired glucose tolerance, central obesity, elevated blood pressure, high triglyceride levels, and low high-density lipoprotein cholesterol levels. Among these metabolic disturbances, impaired glucose tolerance and elevated blood pressure are important causes of CKD. In addition, previous studies have demonstrated that impaired glucose tolerance and diabetes mellitus (DM) can induce renal hyperfiltration, and that they are associated with the development to CKD [9–11]. Furthermore, hypertension (HTN) of all ranges (including prehypertension) is associated with CKD, and patients with CKD are also at increased risk of developing DM or HTN [12–16]. Furthermore, MetS and CKD can induce vascular conditions, such as macro- and micro-angiopathies, including atherosclerosis [5,17].

Several studies have reported an association between each component of the cardiometabolic risk factors and hearing loss [18–21]. However, very few studies have investigated the association between MetS as a broad spectrum syndrome (including the five cardiometabolic risk factors) and hearing loss, or the additive effect on hearing loss according to the number of cardiometabolic risk factors. In addition, CKD is a well-known risk factor for atherosclerosis, and animal studies have reported an association between uremia and cochlear dysfunction [22,23]. However, there are also few studies regarding the association between CKD and hearing loss. Regarding the increase in the burden of MetS and CKD, identifying the association between hearing loss and MetS or CKD will be useful in planning for the evaluation and management of hearing loss in patients with MetS or CKD. Therefore, the aim of this study was to determine whether MetS or CKD is associated with hearing thresholds in the general Korean population.

## Patients and Methods

### Study population

Data from the Korean National Health and Nutrition Examination Survey (KNHANES) 2009–2012 were used for the analyses. KNHANES is a nationwide, multi-stage stratified survey of a representative sample of the entire South Korean population that is conducted by the Korea Centers for Disease Control and Prevention. The total number of participants in KNHANES was 36,067. Participants were excluded from the present study based on lack of data regarding renal function (n = 4,540) or hearing thresholds (n = 4,583), external or middle ear disease (n = 836) or brain disorders such as stroke (n = 1,330), or age <18 years old (n = 8,224). As a result, 16,554 participants were included in this study. This study design was approved by the institutional review board of Yeungnam University Hospital (YUH-14–0487-O71). The board waived the need for informed consent, as the subjects’ records and information were anonymized and de-identified prior to the analysis.

### Study variables

Clinical and laboratory data were collected from the subjects during the health examinations, including age, sex, body mass index (BMI, kg/m^2^), waist circumference (cm), serum creatinine levels (mg/dL), systolic blood pressure (SBP, mmHg), diastolic blood pressure (DBP, mmHg), fasting glucose levels (mg/dL), total cholesterol levels (mg/dL), high-density lipoprotein (HDL) cholesterol levels (mg/dL), triglyceride levels (mg/dL), smoking behavior, alcohol consumption, and estimated glomerular filtration rate (eGFR, mL/min/1.73 m^2^).

DM was defined as a self-reported diagnosis of DM or a fasting glucose level of ≥126 mg/dL. HTN was defined as a SBP of ≥140 mmHg, a DBP of ≥90 mmHg, a self-reported history of HTN, or the use of anti-hypertension medication. BMI was calculated as weight divided by height squared, and MetS was defined using the National Cholesterol Education Program Adult Treatment Panel III guidelines [24]. Serum creatinine levels were measured using a Hitachi Automatic Analyzer (alkaline picrate, Jaffé kinetic). The eGFR was calculated using the Chronic Kidney Disease Epidemiology Collaboration equation [25]. CKD was defined as an eGFR of <60 mL/min/1.73 m^2^ or a dipstick proteinuria result of ≥1+. Smoking behavior was classified as current smoker, ex-smoker, or non-smoker. High-risk alcohol consumption was defined as ≥7 glasses per day for men and ≥5 glasses per day for women. Alcohol consumption was classified into 4 groups according to the frequency of high-risk drinking: never, once a month, once a week, and every day. The homeostatic model assessment for insulin resistance (HOMA-IR) was calculated using the following equation:

HOMA-IR(mg/dL)=[fasting glucose(mg/dL)×fasting insulin(mIU/mL)]/405

Ear examinations and pure-tone audiometry were performed as previously described [26]. None of these subjects were currently receiving medication that is associated with ototoxicity. Histories of explosive or occupational noise exposure were classified as positive or negative, according to the subjects’ recall. An explosive noise was defined as a sudden loud noise, such as an explosion or gunshot. Exposure to occupational noise was determined according to whether the participants had worked in a location with loud machinery for ≥3 months. Loud noise was defined by whether the participants had needed to raise his or her voice to have a conversation. The hearing thresholds were measured at 0.5, 1, 2, 3, 4, and 6 kHz. In the present study, if the hearing threshold of the left ear was higher than that of the right ear, we used only the left ear data (High-frequency (Freq): 29.7 ± 23.8 dB and 30.6 ± 24.3 dB in the right and left ear, respectively; *P* < 0.001). Low-Freq was defined as pure-tone averages at 0.5 and 1 kHz, and Mid-Freq and High-Freq were defined as average thresholds at mid-frequency (2 and 3 kHz) and high frequency (4 and 6 kHz), respectively.

### Statistical analyses

The data were analyzed using SPSS version 19 (SPSS, Chicago, IL, USA). Continuous variables were expressed as mean ± standard deviation, and were compared using *t* tests or one-way analysis of variance, followed by a post-hoc Tukey comparison. Categorical variables were expressed as counts and percentages, and were compared using the Pearson χ^2^ test or Fisher exact test. Correlation analysis was performed to assess the strength of the relationship between continuous variables. Multivariate analyses using analyses of covariance or multiple regression analyses were used to determine the independent predictors of hearing thresholds. For MetS, the covariates were age, smoking, alcohol consumption, BMI, exposure to explosive noise, and exposure to occupational noise. For CKD, the covariates were age, smoking, alcohol consumption, BMI, DM, HTN, exposure to explosive noise, and exposure to occupational noise. The data for the multivariate analyses were expressed as mean ± standard error, and the level of statistical significance was set at *P* < 0.05.

## Results

### Clinical characteristics of the participants

The mean age was 50.4 ± 16.6 years for men (n = 6,741) and 49.2 ± 16.4 years for women (n = 9,813) (Table 1). BMI, waist circumference, serum creatinine levels, SBP, DBP, fasting glucose levels, and triglyceride levels were higher in the men than in the women. However, total cholesterol levels, HDL cholesterol levels, and eGFR were higher in the women than in the men. The prevalence of comorbidities, such as HTN, DM, MetS, and CKD, and heavy alcohol consumption was higher in the men than in the women. The hearing thresholds for men in the Mid- and High-Freq categories were significantly higher than the hearing thresholds for women (*P* < 0.001).

**Table 1:** Clinical characteristics of the participants.

Characteristics	All (n = 16,554)	Male (n = 6,741)	Female (n = 9,813)	*P* value*
Age (years)	49.7 ± 16.5	50.4 ± 16.6	49.2 ± 16.4	<0.001
Body mass index (kg/m^2^)	23.6 ± 3.4	24.0 ± 3.2	23.4 ± 3.5	<0.001
Waist circumference (cm)	80.9 ± 10.0	84.5 ± 9.0	78.4 ± 9.9	<0.001
Serum creatinine (mg/dL)	0.82 ± 0.22	0.97 ± 0.18	0.72 ± 0.18	<0.001
Systolic blood pressure (mmHg)	121.2 ± 18.3	124.1 ± 16.8	119.2 ± 19.0	<0.001
Diastolic blood pressure (mmHg)	76.5 ± 10.8	79.2 ± 10.9	74.6 ± 10.3	<0.001
Fasting glucose (mg/dL)	97.5 ± 22.1	100.3 ± 24.0	95.6 ± 20.5	<0.001
Total cholesterol (mg/dL)	188.5 ± 36.4	186.6 ± 36.0	189.9 ± 36.6	<0.001
Triglyceride (mg/dL)	129.0 ± 99.7	151.5 ± 122.8	113.5 ± 76.3	<0.001
HDL cholesterol (mg/dL)	52.6 ± 12.7	49.3 ± 12.0	54.8 ± 12.7	<0.001
eGFR (mL/min/1.73 m^2^)	94.9 ± 17.5	91.3 ± 16.7	97.4 ± 17.6	<0.001
Hypertension (%)	5,061 (30.6%)	2,401 (35.6%)	2660 (27.1%)	<0.001
Diabetes mellitus (%)	1,689 (10.2%)	859 (12.7%)	830 (8.5%)	<0.001
MetS (%)	4,618 (27.9%)	1,904 (28.3%)	2714 (27.7%)	<0.001
Chronic kidney disease (%)	656 (4.0%)	330 (4.9%)	326 (3.3%)	<0.001
High-risk alcohol consumption				<0.001
Never	8,901 (53.8%)	2354 (34.9%)	6547 (67.0%)	
< one per a month	2,672 (16.1%)	1072 (15.9%)	1600 (16.4%)	
Once a month	2,018 (12.2%)	1136 (16.9%)	882 (9.0%)	
Once a week	2,122 (12.8%)	1540 (22.8%)	582 (6.0%)	
Every day	660 (4.0%)	561 (8.3%)	99 (1.0%)	
No data	144 (0.9%)	78 (1.2%)	66 (0.7%)	
Smoking				<0.001
Non-smoker	10,053 (60.7%)	1335 (88.8%)	8718 (88.8%)	
Ex-smoker	2,714 (16.4%)	2285 (33.9%)	429 (4.4%)	
Current smoker	3,611 (21.8%)	3048 (45.2%)	563 (5.7%)	
No data	176 (1.1%)	73 (1.1%)	103 (1.0%)	
Occupational noise				<0.001
Exposure	1,530 (9.2%)	968 (14.4%)	562 (5.7%)	
Non-exposure	12,076 (72.9%)	4,559 (67.6%)	7,517 (76.6%)	
No data	2948 (17.8%)	1,214 (18.0%)	1,734 (17.7%)	
Explosive noise				<0.001
Exposure	2,651 (16.0%)	2,346 (34.8%)	305 (3.1%)	
Non-exposure	10,932 (66.0%)	3,176 (47.1%)	7,756 (79.0%)	
No data	2,971 (17.9%)	1,219 (18.1%)	1,752 (17.9%)	
Low-Freq (dB)	14.9 ± 15.0	15.1 ± 14.5	14.9 ± 15.3	0.367
Mid-Freq (dB)	18.9 ± 19.6	22.2 ± 21.3	16.6 ± 18.0	<0.001
High-Freq (dB)	30.6 ± 24.3	37.3 ± 26.5	26.1 ± 21.5	<0.001

Data are expressed as numbers (percentages) for categorical variables and mean ± standard deviation for continuous variables.

*P values between males and females were tested using the *t*-test for continuous variables and the Pearson χ^2^ test or Fisher exact test for categorical variables.

Abbreviations: HDL, high-density lipoprotein; MetS, metabolic syndrome; Low-Freq, low frequency; Mid-Freq, mid-frequency; High-Freq, high-frequency; dB, decibel.

### Association between hearing thresholds and metabolic syndrome or CKD

**Fig 1:**
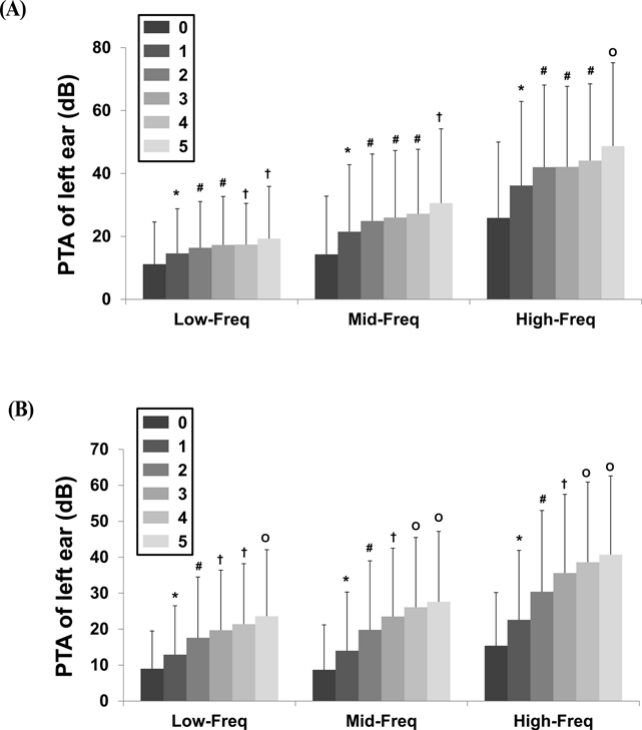
Hearing thresholds according to number of metabolic syndrome components. A. Men B. Women (P < 0.001 for trend in both sexes). Data in men are: Low-Freq, 11.2 ± 13.4 dB with 0, 14.6 ± 14.2 dB with 1, 16.4 ± 14.7 dB with 2, 17.3 ± 15.4 dB with 3, 17.4 ± 13.1 dB with 4, and 19.3 ± 16.6 dB with 5; Mid-Freq, 14.3 ± 18.5 dB with 0, 21.5 ± 21.3 dB with 1, 24.9 ± 21.3 dB with 2, 26 ± 21.3 dB with 3, 27.2 ± 20.5 dB with 4, and 30.6 ± 23.6 dB with 5; High-Freq, 25.9 ± 24.1 dB with 0, 36.2 ± 26.7 dB with 1, 42 ± 26.1 dB with 2, 42.1 ± 25.6 dB with 3, 44.1 ± 24.4 dB with 4, and 48.7 ± 26.5 dB with 5. Data in women: Low-Freq, 9.0 ± 10.5 dB with 0, 12.9 ± 13.6 dB with 1, 17.6 ± 16.9 dB with 2, 19.7 ± 16.7 dB with 3, 21.4 ± 16.8 dB with 4, and 23.6 ± 18.5 dB with 5; Mid-Freq, 8.7 ± 12.5 dB with 0, 14 ± 16.3 dB with 1, 19.8 ± 19.2 dB with 2, 23.5 ± 19 dB with 3, 26.1 ± 19.4 dB with 4, and 27.6 ± 19.6 dB with 5; High-Freq, 15.4 ± 14.8 dB with 0, 22.6 ± 19.3 dB with 1, 30.4 ± 22.6 dB with 2, 35.6 ± 21.9 dB with 3, 38.6 ± 22.3 dB with 4, and 40.7 ± 21.9 dB with 5. *P < 0.05 compared to participants with 0. ^#^P < 0.05 compared to participants with 0 or 1. ^†^P < 0.05 compared to participants with 0, 1, or 2. ^O^P < 0.05 compared to participants 0, 1, 2, or 3. Abbreviations: PTA, pure tone audiometry; Low-Freq, low-frequency; Mid-Freq, mid-frequency; High-Freq, high-frequency; dB, decibel.

Male and female subjects with MetS had higher hearing thresholds than subjects without MetS (Table 2). In men, the mean Low-Freq thresholds according to the number of MetS components were 11.2 ± 13.4 dB, 14.6 ± 14.2 dB, 16.4 ± 14.7 dB, 17.3 ± 15.4 dB, 17.4 ± 13.1 dB, and 19.3 ± 16.6 dB for 0–5 MetS components, respectively (Fig. 1A). In women, the mean Low-Freq thresholds were 9.0 ± 10.5 dB, 12.9 ± 13.6 dB, 17.6 ± 16.9 dB, 19.7 ± 16.7 dB, 21.4 ± 16.8 dB, and 23.6 ± 18.5 dB for 0–5 MetS components, respectively (Fig. 1B). The trends for the Mid- and High-Freq thresholds according to the number of MetS components were similar to those observed for the Low-Freq thresholds. All three hearing thresholds increased as the number of MetS components increased.

**Table 2:** Difference in hearing thresholds according to the presence of metabolic syndrome.

Characteristics	MetS (-)	MetS (+)	*P* value
Male			
Low-Freq (dB)	14.1 ± 14.3	17.5 ± 14.9	<0.001
Mid-Freq (dB)	20.3 ± 21.0	26.8 ± 21.3	<0.001
High-Freq (dB)	34.9 ± 26.5	43.3 ± 25.4	<0.001
Female			
Low-Freq (dB)	12.6 ± 13.9	20.8 ± 17.0	<0.001
Mid-Freq (dB)	13.4 ± 16.4	24.9 ± 19.3	<0.001
High-Freq (dB)	21.8 ± 19.6	37.3 ± 22.1	<0.001

Data are expressed as mean ± standard deviation.

*P values between MetS (-) and MetS (+) were tested using the *t*-test.

Abbreviations: MetS (-), absence of metabolic syndrome; MetS (+), presence of metabolic syndrome; Low-Freq, low-frequency; Mid-Freq, mid-frequency; High-Freq, high-frequency; dB, decibel.

**Fig 2:**
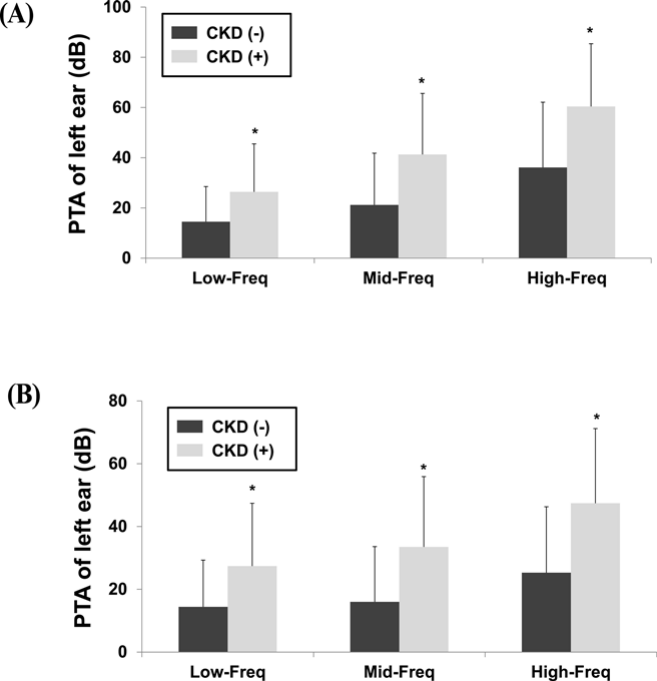
Hearing thresholds according to the presence of chronic kidney disease. A. Men B. Women (*P < 0.001 versus absence of chronic kidney disease). Abbreviations: CKD, chronic kidney disease; PTA, pure tone audiometry; Low-Freq, low frequency; Mid-Freq, mid-frequency; High-Freq, high-frequency; dB, decibel.

**Fig 3:**
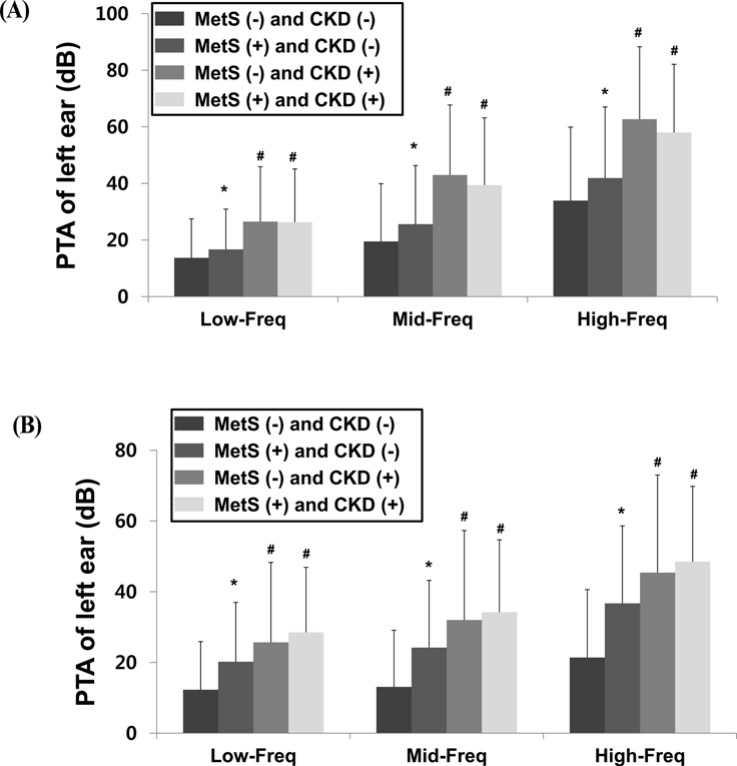
Influence on hearing threshold according to the presence of metabolic syndrome (MetS) and/or chronic kidney disease (CKD). A. Men B. Women (*P < 0.05 compared to subjects with neither MetS nor CKD, ^#^ *P* < 0.05 compared to subjects with neither MetS nor CKD, and to subjects with only MetS). Abbreviations: CKD, chronic kidney disease; MetS, metabolic syndrome; PTA, pure tone audiometry; Low-Freq, low-frequency; Mid-Freq, mid-frequency; High-Freq, high-frequency; dB, decibel.

The hearing thresholds were significantly higher in men with CKD than those in men without CKD (Low-Freq: 26.4 ± 19.1 dB vs. 14.5 ± 14.0 dB; Mid-Freq: 41.3 ± 24.3 dB vs. 21.2 ± 20.6 dB; High-Freq: 60.4 ± 25.0 dB vs. 36.1 ± 26.0 dB, respectively; *P* < 0.001) (Fig. 2A). Similarly, the hearing thresholds were significantly higher in women with CKD than those in women without CKD (Low-Freq: 27.4 ± 20.0 dB vs. 14.4 ± 14.9 dB; Mid-Freq: 33.5 ± 22.4 dB vs. 16 ± 17.6 dB; High-Freq: 47.4 ± 23.8 dB vs. 25.3 ± 21.0 dB, respectively; *P* < 0.001) (Fig. 2B). Among the male subjects, 4,661 subjects had neither MetS nor CKD, 1,743 had only MetS, 169 had only CKD, and 161 had both MetS and CKD. Among the female subjects, 6,967 subjects had neither MetS nor CKD, 2,508 had only MetS, 119 had only CKD, and 206 had both MetS and CKD. However, 7 men and 13 women were excluded due to a lack of data. Men and women with only MetS had higher hearing thresholds than the subjects with neither MetS nor CKD (Fig. 3A-B). Subjects with only CKD had higher hearing thresholds than those with neither MetS nor CKD. However, there were no significant differences in the hearing thresholds of subjects with only CKD and those with both MetS and CKD. These findings indicate that MetS does not have an additive effect among subjects with CKD.

Table 3 shows the correlation between the HOMA-IR or eGFR values and the hearing thresholds. In men, the correlation coefficients for eGFR were-0.352, -0.446, and-0.478 for Low-, Mid- and High-Freq, respectively. In women, the correlation coefficients for eGFR were-0.401, -0.478, and-0.527 for Low-, Mid-, and High-Freq, respectively.

**Table 3:** Correlation between eGFR or HOMA-IR values and hearing thresholds.

	Male		Female	
	*r*	*P* value	*r*	*P* value
eGFR vs Hearing threshold				
Low-Freq	-0.352	<0.001	-0.401	<0.001
Mid-Freq	-0.446	<0.001	-0.478	<0.001
High-Freq	-0.478	<0.001	-0.527	<0.001
HOMA-IR vs Hearing threshold				
Low-Freq	0.033	0.063	0.103	<0.001
Mid-Freq	0.034	0.055	0.108	<0.001
High-Freq	0.025	0.157	0.127	<0.001

Abbreviations: eGFR, estimated glomerular filtration rate; HOMA-IR, homeostasis model assessment of insulin resistance; *r*, correlation coefficient; Low-Freq, low-frequency; Mid-Freq, mid-frequency; High-Freq, high-frequency.

HOMA-IR was 2.58 ± 1.74 in men who were <65 years old, 2.60 ± 1.73 in men who were ≥65 years old, 2.33 ± 1.30 in women who were <65 years old, and 2.87 ± 1.90 in women who were ≥65 years old. There was no significant difference in the men’s HOMA-IR for each age group, the HOMA-IR results for elderly women were significantly higher than those for men or younger women. A significant correlation between HOMA-IR and the hearing thresholds was only observed in women.

Table 4 shows that MetS was associated with decreased hearing thresholds in women, and CKD was associated with decreased hearing thresholds in men and women. Multiple regression analysis shows similar results, with the exception of Low-Freq in women (Table 5).

**Table 4:** Multivariate analyses for hearing thresholds according to the presence of metabolic syndrome or chronic kidney disease.

	MetS (-)	MetS (+)	*P*-value*	CKD (-)	CKD (+)	*P*-value*
Male						
Low-Freq	14.6 ± 0.2	14.9 ± 0.3	0.416	14.6 ± 0.2	19.0 ± 0.8	<0.001
Mid-Freq	21.9 ± 0.3	22.2 ± 0.4	0.547	21.9 ± 0.2	27.5 ± 1.0	<0.001
High-Freq	37.1 ± 0.3	37.1 ± 0.5	0.959	37.1 ± 0.3	41.3 ± 1.2	<0.001
Female						
Low-Freq	14.3 ± 0.2	15.1 ± 0.3	0.030	14.5 ± 0.1	16.2 ± 0.2	0.032
Mid-Freq	15.9 ± 0.2	16.9 ± 0.3	0.011	16.2 ± 0.2	18.6 ± 0.8	0.004
High-Freq	25.2 ± 0.2	26.8 ± 0.4	0.001	25.7 ± 0.2	28.0 ± 0.9	0.015

Data are expressed as mean ± standard error.

*Statistical significances between the presence and absence of MetS or CKD were calculated.

Covariates for MetS were age, smoking, alcohol consumption, body mass index, exposure to explosive noise, and exposure to occupational noise. Covariates for CKD were age, smoking, alcohol consumption, body mass index, diabetes mellitus, hypertension, exposure to explosive noise, and exposure to occupational noise.

Abbreviations: MetS, metabolic syndrome; CKD, chronic kidney disease; Low-Freq, low frequency; Mid-Freq, mid-frequency; High-Freq, high-frequency.

**Table 5:** Regression analyses for hearing thresholds according to the presence of metabolic syndrome or chronic kidney disease.

	Univariate				Multivariate			
	Male		Female		Male		Female	
	St-β ± SE	*P* value	St-β ± SE	*P* value	St-β ± SE	*P* value	St-β ± SE	*P* value
Low-Freq								
MetS	0.105 ± 0.391	<0.001	0.241 ± 0.335	<0.001	0.011 ± 0.405	0.416	0.025 ± 0.373	0.030
CKD	0.177 ± 0.806	<0.001	0.153 ± 0.851	<0.001	0.061 ± 0.773	<0.001	0.018 ± 0.804	0.073
Mid-Freq								
MetS	0.136 ± 0.570	<0.001	0.286 ± 0.389	<0.001	0.007 ± 0.542	0.547	0.027 ± 0.404	0.011
CKD	0.204 ± 1.175	<0.001	0.173 ± 1.000	<0.001	0.053 ± 1.035	<0.001	0.021 ± 0.871	0.020
High-Freq								
MetS	0.143 ± 0.709	<0.001	0.324 ± 0.459	<0.001	-0.001 ± 0.635	0.959	0.033 ± 0.451	0.001
CKD	0.199 ± 1.464	<0.001	0.184 ± 1.190	<0.001	0.033 ± 1.215	0.001	0.017 ± 0.974	0.045

Covariates for MetS were age, smoking, alcohol consumption, body mass index, exposure to explosive noise, and exposure to occupational noise. Covariates for CKD were age, smoking, alcohol consumption, body mass index, diabetes mellitus, hypertension, exposure to explosive noise, and exposure to occupational noise.

Abbreviations: MetS, metabolic syndrome; CKD, chronic kidney disease; Low-Freq, low-frequency; Mid-Freq, mid-frequency; High-Freq, high-frequency; St-β, standardized- β; SE, standard error.

## Discussion

Previous studies have shown that men have poorer hearing thresholds compared to women and that the left ear has a consistently worse hearing threshold than the right ear [27]. Thus, we analyzed data for men and women from KHANES 2009–2012, and defined the hearing thresholds using only data from the left ear. Our results indicate that women with MetS had higher hearing thresholds than those without MetS. In addition, the number of MetS components and the presence of insulin resistance were associated with the hearing thresholds. We analyzed eGFR as a continuous variable and CKD as categorical variable, and both were associated with the hearing thresholds in men and women.

MetS is a well-known condition that is linked to the development of various complications, such as DM, HTN, dyslipidemia, and cardiovascular disease [5,6,17]. In addition, Bainbridge KE et al. have recently reported an association between hearing thresholds and DM, angiopathy, dyslipidemia, and neuropathy [21,28,29]. Another study has reported that the underlying mechanism for the association between DM and presbycusis may be related to oxidative stress and the deposition of advanced glycation end-products [30]. Based on these findings, we hypothesized that the associations between MetS and hearing may be similar to the association observed between DM and hearing.

In the present study, there was a significant association between MetS or HOMA-IR and hearing thresholds in women. MetS is a cause and result of insulin resistance, while HOMA-IR is a well-known index of insulin resistance. Our results revealed an association between these markers for insulin resistance and hearing thresholds in women, although not in men. While the cause for this gender-related difference is not yet clear, changes in estrogen and adiponectin levels may be relevant. For example, estrogen plays a major role in the auditory system, and baseline adiponectin levels are higher in women than in men [31,32]. However, central obesity is rapidly increased among elderly women, who also experience a decrease in estrogen and adiponectin levels. Therefore, these decreases in adiponectin and estrogen levels may be associated with the increase in cochlear damage due to metabolic disturbances. Hwang et al. have shown that the association between adiponectin levels and hearing thresholds only exists among elderly women [32]. In present study, the HOMA-IR results for elderly women were significantly higher than those for men or younger women. In addition, HOMA-IR was correlated with hearing threshold among the women. Therefore, further investigations are needed to identify the cause of this gender-related difference.

Although the underlying mechanisms for the association between CKD and hearing are not clear, uremic neuropathy and fluid or electrolyte abnormalities are two possible causes [22,23,33]. CKD patients can present with complicated, variable neuropathies, such as mono-, poly-neuropathy, and encephalopathy [34]. These are more likely to develop when CKD progresses end-stage renal disease, and most cases require renal replacement therapies. Antonelli et al. have also shown that uremia is associated with abnormal waveforms during auditory brainstem response audiometry [33]. In addition, the kidney and cochlear have similar structures, such as ciliated epithelial cells and tubular structures, and similar physiological mechanisms for the transport of fluids and electrolytes (e.g., aquaporin and Na^+^, K^+^-ATPase); these mechanism are sensitive to blood pressure, blood supply, and vasoconstriction [35–39]. Therefore, CKD and hearing loss actually share multiple risk factors, such as age, DM, and HTN. Several observational studies have reported an association between CKD and hearing loss, although the number of included patients was small. In addition, most studies have enrolled patients who have reached end-stage renal disease or who have underwent renal replacement therapies [40–42]. Vilayur et al. have used categorical criteria for hearing loss (average pure-tone threshold >25dB) to demonstrate that CKD was associated with the development of hearing loss [43]. Although their study included a relatively large sample, it only evaluated the association between CKD and hearing using data from a homogeneous Australian population (no ethnic differences). In addition, their study did not show data for the hearing thresholds.

The present study is the largest cross-sectional analysis to demonstrate the association between hearing threshold and CKD in an Asian population. However, it also has a number of limitations. First, the cross-sectional study design prevented us from evaluating the changes in hearing levels. Therefore, prospective analysis is needed to confirm whether there is a casual relationship between changes in renal function and hearing thresholds. Second, this study did not include an analysis of causative markers, such as adipokines, oxidative stress, or vasculopathy, which are known to be associated with the development of hearing impairment in MetS or CKD. Third, this study did not evaluate any sensitive components of hearing difficulties, such as speech discrimination. Fourth, CKD was defined as an eGFR of <60 mL/min/1.73 m^2^ or a dipstick proteinuria result of ≥1+, and creatinine and proteinuria were evaluated using a single measurement. Unfortunately, repeated measurements are needed for a definite diagnosis, and these measurements were not performed in the present study. However, many studies in large-scale populations have analyzed results from a single measurement for proteinuria [44–48]. We believe that the impact of these limitations is reduced by the large sample size in the present study.

In conclusion, MetS is associated with hearing thresholds in women, and CKD is associated with hearing thresholds in men and women. Therefore, patients with MetS or CKD should be closely monitored for hearing impairment.
